# Establishment of a Gene Detection System for Hotspot Mutations of Hearing Loss

**DOI:** 10.1155/2018/6828306

**Published:** 2018-03-07

**Authors:** Chao Wang, Shengzhou Wang, Hongyan Chen, Daru Lu

**Affiliations:** State Key Laboratory of Genetic Engineering, School of Life Science, Zhongshan Hospital, Fudan University, 2005 Songhu Road, Shanghai 200438, China

## Abstract

Hearing loss is an etiologically heterogeneous trait with a high incidence in China. Though conventional newborn hearing screening program has been widely adopted, gene detection can significantly improve the means of early discovering genetic risk factors. Thus, simple and efficient methods with higher sensitivity and lower cost for detecting hotspot mutations of hearing loss are urgently requested. Here we established a mutation detection system based on multiple fluorescent probe technique, which can detect and genotype nine hotspot mutations of four prominent hearing loss-related genes in two reactions on a four-channel real-time PCR instrument, including* GJB2* (rs750188782, rs80338943, rs1110333204, and rs80338939),* GJB3* (rs74315319),* SLC26A4* (rs111033313 and rs121908362), and* mtDNA 12S rRNA* (rs267606617 and rs267606619). This system is with high sensitivity that enables detecting as low as 10 DNA copies samples per reaction. A comparison study in 268 clinical samples showed that the detection system had 100% concordance to Sanger sequencing. Besides, blood and saliva samples can be directly detected without DNA extraction process, which greatly simplifies the manipulation. The new system with high sensitivity, accuracy, and specimen type compatibility can be expectedly a reliable tool in clinical application.

## 1. Introduction

Hearing loss, especially nonsyndromic hearing loss (NSHL), is an etiologically heterogeneous trait caused by many known genetic and environmental elements [1]. In China, there are about 0.8 million children under 7 years old whose hearing is impaired, and this number is growing with over 30,000 newly deaf children every year [2]. More than half of them is attributed to the genetic factors and approximately 70% are nonsyndromic hearing loss.

Newborn audiological hearing screening has been widely adopted and has achieved a certain degree of success. However, among newborns who have passed hearing screening, there are still some children carrying one or more risk alleles or mitochondrial mutations. Many studies have reported that, in most cases, damage caused by hearing loss in infancy is irreversible, affecting the development of cognitive, language ability and social acceptability [3]. Accumulation of large epidemiological studies indicated that the risk for hearing loss was extremely high with the hotspots mutations in* GJB2*,* SLC26A4*,* GJB3*, or* mtDNA 12S rRNA* [4–7]. In addition, previous studies indicated that the conventional newborn hearing screening program could be greatly improved by adding a genetic component [1, 2]. Gene detection can complement the current newborn hearing screening program and provides additional insights beyond what conventional audiological tests reveal, thus significantly advancing the current practice for improving newborn hearing screening as a means of early discovering of genetic risk factors.

So far, various techniques are used to screen mutations and some already used for hearing loss-related gene mutations, such as traditional allele specific oligonucleotide analysis, including restriction fragment length polymorphism (RFLP) [8, 9] and single strand conformational polymorphism (SSCP) [10], denaturing high-performance liquid chromatography (DHPLC) [11], amplification refractory mutation system quantitative PCR (ARMS-PCR) analysis [12, 13], microarray-based hybridization biochip assay [14, 15], the next-generation sequencing (NGS), and Sanger sequencing. However, most of these methods are low-throughput, time-consuming, and expensive for multiple gene detection. Although direct DNA sequencing is considered as gold standard for identifying mutations, it remains laborious and time-consuming. These problems have greatly hindered the clinical popularization of newborn hearing loss gene detection [16].

Currently, gene detection method based on multiple fluorescent probe technique combined with melting curve analysis has been developed to many successful assays. The accuracy, ease-of-use, rapidity, and cost-effectiveness have allowed the increasing development in screening for risk factors, typing of pathogen, and detection of genetic disease predisposition [17–22].

In this study, we established a new multiplex system based on fluorescent probe technique that allows the simultaneous detecting and genotyping of nine hotspot mutations of four prominent hearing loss-related genes,* GJB2* (rs750188782, rs80338943, rs1110333204, and rs80338939),* GJB3* (rs74315319),* SLC26A4* (rs111033313 and rs121908362), and* mtDNA 12S rRNA* (rs267606617 and rs267606619) in two reactions by a four-channel real-time PCR instrument. After amplification, the genotyping results of nine hotspot mutations were gained by the melting curve analysis. Allelic types of each mutation are all individually identified by their *Tm* values, labeling fluorophores, and the reaction names. The established system was evaluated regarding the sensitivity, accuracy, and dynamic ranges with different qPCR platforms.

## 2. Materials and Method

### 2.1. Sample Preparation

Human whole blood samples were obtained from Tongji University affiliated Shanghai Pulmonary Hospital. Genomic DNA was extracted separately using TIANamp Genomic DNA Kit (Tiangen, China) according to manufacturer's instructions. DNA was diluted in ddH_2_O and then measured by the absorbance at 260 nm (NanoDrop 2000; Thermo Fisher Scientific) and normalized to the final concentration 20 ng/*μ*L. In addition, human whole blood sample was spotted on the FTA™ sample collection cards (GE Healthcare), allowed to dry at ambient temperature for 0.5 h and stored in a cool dry place. FTA cards should be sampled within 1 week of spotting. Human saliva samples were obtained from Shanghai ZhongYou Biological Technology Co., Ltd. The saliva samples were collected by specimen collection flocked swabs (HR, Jiangxi Haorui) and then prepared with pyrolysis solution (10 mM Tris-HCl pH 8.0, 2 mM EDTA, 200 mM NaCl and 3% SDS) and proteinase K through a briefly pyrolysis process according to the following program: 65°C for 30 min, 95°C for 10 min.

### 2.2. Standard Plasmids Construction

The standard plasmids containing the respective types of nine sites were constructed on the basis of the type-specific sequences provided by the Genome Sequencing Project in GeneBank (http://www.ncbi.nlm.nih.gov/) and confirmed by sequencing. The plasmids were extracted using TIANprep Mini Plasmid Kit (TiangenBiotech, China) according to the instructions of the manufacturer. The concentration of each plasmid was determined by NanoDrop 2000. To determine the limit of detection (LOD) of the real-time PCR assay, the plasmids were diluted to a series of concentrations: 10^10^, 10^9^, 10^8^, 10^7^, 10^6^, 10^5^, 10^4^, 10^3^, 10^2^, and 10^1^ copies/*μ*L. These plasmid preparations were also used to study the reproducibility of the melting temperature (*Tm*) measurement and the specificity of mutation sites genotyping.

### 2.3. Primers and Probes

The primers and probes were designed by Primer Premier 3.0 and The UNAFold Web Server (http://unafold.rna.albany.edu/); all the primers and probes were synthesized and purified by Sango Biotech (Shanghai, China). The two reactions of our system were called Tube-A and Tube-B, respectively. Tube-A, covering five sites, is rs74315319, rs111033313, rs121908362, rs267606617, and rs267606619, and four pairs of primers were designed to amplify them. Tube-B, covering four sites, is rs750188782, rs80338943, rs1110333204, and rs80338939, and two pairs of primers were designed to amplify them. Multiple targets of each reaction can be amplified with a pair of universal primers designed for asymmetric amplification. The primers sequences are shown in Table 1.

A total of nine dually labeled fluorescence probes were designed for the nine respective mutation sites. The probes of rs74315319, rs111033313, and rs750188782 were labeled with 5′-FAM and 3′-black hole quencher 1 (BHQ1), the probes of rs121908362 and rs80338943 were labeled with 5′-ROX and 3′-black hole quencher 2 (BHQ2), the probes of rs267606617 and rs1110333204 were labeled with 5′-HEX and 3′-black hole quencher 1 (BHQ1), and the probes of rs267606619 and rs80338939 were labeled with 5′-Cy5 and 3′-black hole quencher 3 (BHQ3). Each probe can detect two types of site. The probe sequences are shown in Table 2.

### 2.4. PCR and Melting Curve Analysis

The reactions were performed in a 20 *μ*l solution containing HpH buffer [23], 3.5 mM MgCl_2_, 0.25 mM dNTPs, 1 U HS Taq DNA polymerase (TaKaRa, Dalian, China), 0.1 *μ*M probe, 0.05 *μ*M (Tube-A) or 0.2 *μ*M (Tube-B) of each specific primer, 0.125 *μ*M (Tube-A) or 0.2 *μ*M (Tube-B) forward universal primer, 1.25 *μ*M (Tube-A) or 2 *μ*M (Tube-B) reverse universal primer, 2 *μ*L aqueous template (extracted genomic DNA or pyrolysis saliva), or a 1.0 mm Harris manual micropunch (Sangerbio, Shanghai) to punch holes of FTA sample collection cards for sampling dried blood spots; a successful punch was obtained when the FTA disc was transferred from card to target well. Contamination testing has already been undertaken to demonstrate that taking a blank FTA disc between samplings of dried blood spots was sufficient for cleaning the punch heads, ensuring that no DNA carryover occurred during the punching process [24]. When the sample for detection is dried blood spots, 10% (V/V) glycerin should be added [23]. Amplification and melting curve analyses were performed on LightCycler® 96 (Roche) according to the following program: denatured at 95°C for 5 min, 45 cycles of 95°C for 20 s, 60°C for 50 s, and 72°C for 40 s; extension at 72°C for 5 min, 95°C for 3 min; hybridization at 40°C for 3 min, followed by an incremental temperature rise from 40°C (45°C) to 85°C (90°C), 5 readings/°C, at a ramp rate of 0.05°C/s. Fluorescence intensity was measured in four detection channels: FAM, HEX, ROX, and Cy5.

## 3. Results

### 3.1. Typical Genotyping Results of the Standard Plasmids

**Figure 1:**
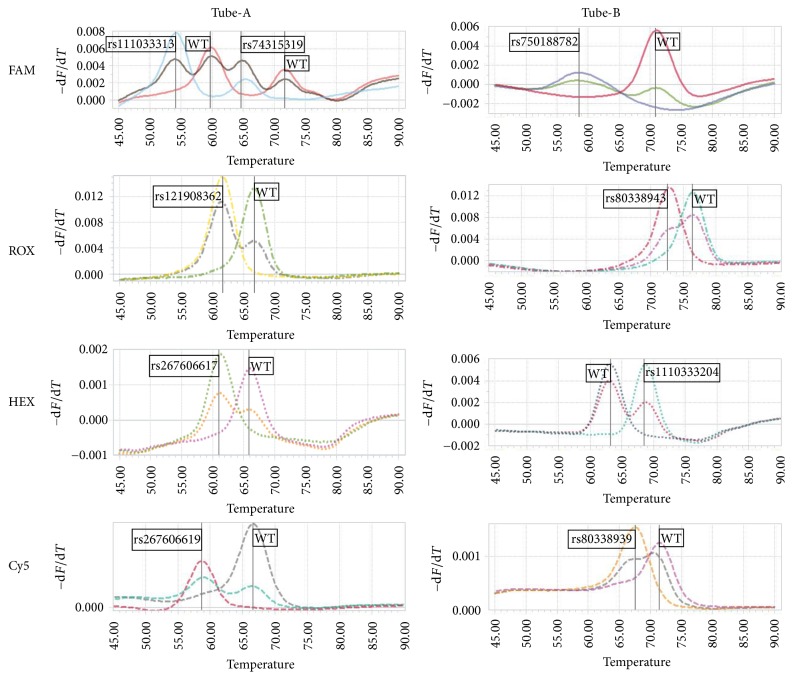
Typical genotyping results of the standard plasmids. The graphic information containing *Tm* values was automatically produced by the software. Melting curves and corresponding genotypes of the nine mutations and wild-type plasmid DNA templates are given according to the four detection channels, shown by the corresponding fluorophores. Tube-A and Tube-B represent the two reactions, respectively.

The established system is a two-reaction, four-channel detection method, based on multiple fluorescent probe technique combined with melting curve analysis. The program contains two stages, amplification and melting, which can be finished in consecutive procedures within 2.5 h on LightCycler 96. The typical results from the standard plasmids on LightCycler 96 are shown in Figure 1. The melting temperature of different genotypes of each site is shown in Table 3.

### 3.2. Detection Sensitivity Assay

**Figure 2:**
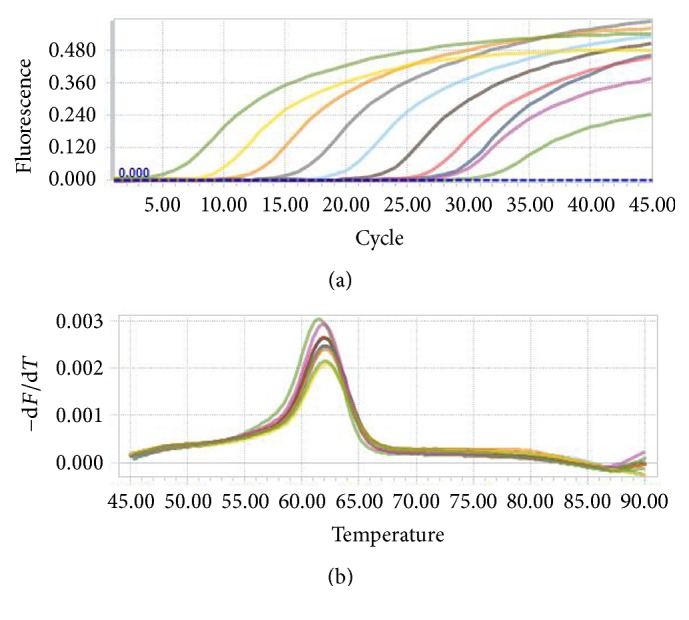
The rs111033313 site detection sensitivity assay result. The concentration of plasmid DNA was 1 × 10^10^ to1 × 10^1^ copies per reaction. (a) Because of the difference in the initial template concentration, the Ct value increased consistently as the template concentration was reduced. (b) The fluorescence intensity of detection assay results is almost the same and did not consistently change with the initial template concentration. It was suggested that the low concentration sample could still yield an ideal detection result.

Using plasmid DNA as the amplification template, the limit of detection (LOD) of the assay was 10 DNA copies per reaction, though the standard curve method the amplification efficiency was 113.6% to 115.9% [23, 25]. A linear relationship between the Ct value and the logarithmic DNA concentration was achieved in the range of 1 × 10^10^ to 1 × 10^1^ copies per reaction (*R*^2^ > 0.98). All mutation sites could be identified by their corresponding *Tm* values. The rs111033313 site detection sensitivity assay result on LightCycler 96 is shown in Figure 2.

### 3.3. Clinical Sample Detection

**Figure 3:**
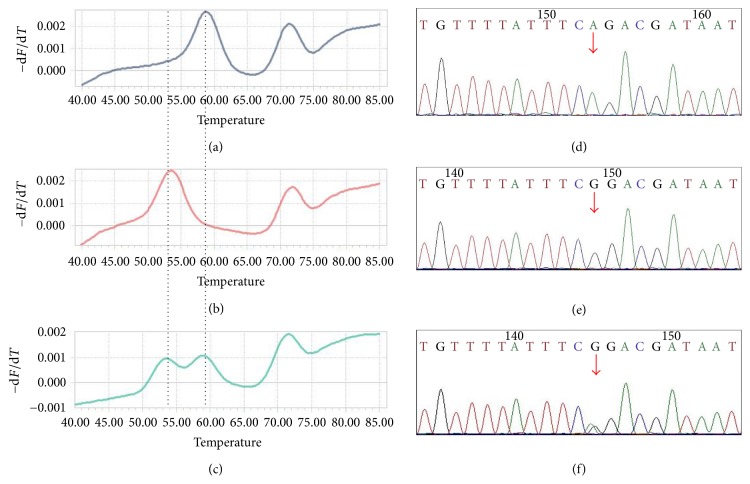
The rs111033313 detection results proved by the Sanger sequence results. Sequence results (d), (e), and (f) correspond to the detection results (a), (b), and (c) respectively. (a) The wild homozygous genotype of the rs111033313 site, (b) the mutant homozygous genotype of the rs111033313 site, and (c) the heterozygote genotype of the rs111033313 site.

The detection was performed with extracted DNA, dried blood spots, and saliva pyrolysis solution. We evaluated our detection system with a double-blind testing of 268 extracted genomic DNA samples; the detection result is shown in Table 4. Finally, the detection results were confirmed by sequencing, which proved the accuracy and specificity of our system. The typical results on LightCycler 96 of the rs111033313 from the DNA samples are shown in Figure 3.

Through comparison, we found that three kinds of samples, extracted genomic DNA, dried blood spots, and saliva pyrolysis solution can all yield ideal detection results, the system can ensure that the fluorescence intensity of the three kinds of samples is almost the same and is able to recognize the different genotypes. However, it is necessary to add 10% glycerin, a typical PCR enhancer, to the dried blood spots sample solution to improve the amplification yield, which will lead to a 2~3°C reduced *Tm* value [26].

### 3.4. Cross-Platform Compatibility Assay

To test the cross-platform compatibility of the system, the detection with the same plasmid samples was performed on three types of commonly used real-time PCR instruments: LightCycler 96 (Roche), LightCycler® 480 (Roche), and CFX-96 (Bio-Rad). The results showed that the detection and genotyping assay could be accomplished on all of the aforementioned platforms. The three types of instruments actually differed in the program for melting curve analysis as well as in the format of data analysis. Manufacturers' recommended melting conditions and the time spent on melting analysis after PCR as shown in Table 5.

Among three different platforms, a systematic *Tm* shift of 0.5 to 4°C was observed in some certain sites; it is probably due to the varied melting programs. However, the *Tm* values obtained from certain instrument proved to be highly reproducible under constant reaction conditions. The *Tm* values obtained from three different platforms are shown in Table 6.

## 4. Discussion

In this study, we applied the gene detection method based on multiple fluorescent probe technique to successfully establish a new gene detection system; it could simultaneously detect and genotype nine hot mutation sites of hearing loss genes in two reactions, enabling multilayer information to be retrieved from a sample with no extra manipulations.

The key feature of our system is the utilization of multiple site-specific probes with certain *Tm* values in two reactions to identify the respective nine mutation sites. Since each mutation needs a specific probe, the number of fluorescence detection channels in a fluorometric thermocycler becomes the bottleneck for multiplex detection. This was addressed by the utilization of the dually labeled, self-quenched probes, which allowed us to design probes with flexible adjusted and well-separated *Tm* values in one channel. On one hand, it ensures that the different mutation sites could be distinguished easily and accurately; it also provides the possibility of achieving the multisite detection in a single channel, on the other hand. We made an attempt in FAM channel in the Tube-A and successfully detected the four alleles of two heterozygote sites at the same time; the four peaks can be shown completely and clearly without *Tm* cross-talk between probes and could possibly cover more regions [27].

Generally, there are three main difficulties in multiplexed PCR: the readout of different amplicons, the depletion of dNTPs by the highest concentration amplicons, and the formation of primer dimers during amplification [28]. In our system, the first difficulty was overcome by the hybridization of the specific dual-labeled, self-quenched probes and the target. To solve the last two difficulties, we proposed asymmetric amplification methods using universal primers. As an end-point detection type method, the detection system requires excess single-stranded amplicon for probe hybridization to generate sufficient fluorescence signal. Asymmetric amplification ensures that the sufficient single strand target can be produced. Primer dimers refer to the unintended interaction between primers and always result in the formation of short amplicons with sequence unrelated to any templates. For single-plex PCR with two primers, careful design and optimization of a primer sequence can result in a good set of primers with little primer dimer formation. However, in multiplex PCR for simultaneous analysis of *N* templates, there are 2*N* primers, which result in at least 4*N*^2^ possible primer dimer interactions. In our system, there are four amplicons (within five sites) to be produced in Tube-A and two amplicons (within four sites) to be produced in Tube-B. Accordingly, we used universal primers to solve the problem of amplification inhomogeneity and interference of the primer dimer. With the universal primers, the concentration of the specific long primers can be reduced greatly, through the first several cycles, it will accumulate a small amount of product, and, after several cycles, the universal primers will begin to amplify the products indiscriminately and yield excess single-stranded product.

The *Tm* values obtained from melting curve analysis proved to be highly reproducible and concordanced under constant reaction conditions on the certain instrument. Among three different platforms we tested, a systematic *Tm* shift of 0.5 to 4°C was observed in some certain sites, due to the varied melting programs. Generally, an identical PCR amplification program can be easily set up in all instruments but the melting curve programs are various from different instruments, as previously reported [19, 29, 30]. Many parameters can affect the *Tm* value of melting curve analysis results, such as ramp rate, acquisitions frequency, and step hold time. Instead of the direct and simple °C/s as the unit of melting rate, various manufacturers have adopted creative but confusing units, such as “*X* acquisitions/°C,” “*X*°C step with a *Y* s hold,” “*X*°C ramp with a *Y* s hold,” or “*X*% ramp.” It was difficult for us to set the identical melting curve program on different platforms and it can be further studied on the setup of the most relevant parameters with *Tm* value.

Traditionally, extracting aqueous DNA as PCR template is required in detection, with the development of noninvasive diagnosis; collection medium has been used in human DNA sampling, processing, and long-term storage for years, and now biological samples such as saliva or blood are collected with FTA sample collection cards and specimen collection flocked swabs become more and more extensive [31, 32]. Accordingly, in our system, we optimize the amplification condition and detection system to make it adapt to the sample collected with the collection medium directly, without a series of complex extraction steps and prevented the biological fluid from degradation, thus allowing for easy transport and long-term storage [33, 34]. Additionally, the two-reaction system could process 48 samples and get the detection result within 2.5 h in a single run on LightCycler 96. Comparing with other aforementioned methods, it can at least save about 1~2 hours on average for the same sample size, leading to significant time saving.

## 5. Conclusion

In summary, we applied the gene detection method based on multiple fluorescent probe technique to establish a new system that allows the simultaneous detecting and genotyping of nine mutation sites of four prominent hearing loss-related genes. The probe-based method ensured the reliability of this system, while the closed-tube operation mode significantly reduced post-PCR manipulations, time requirements, and carryover contamination. We performed the system on the widely available real-time qPCR instrument which would facilitate its acceptance in clinical settings. Furthermore, the easy manipulation, low cost, features of high performance, accuracy, and popularization of the system will make it a perfect prospect in clinical application.

## Figures and Tables

**Table 1:** Primer sequences.

Primer name	Primer sequences (5′→3′)
Univer Primer-F	GTGGCAGGGCGCTACGAACAAT
Univer Primer-R	CATGTGAGATTGGATCTTGCTGGGC
rs74315319-F	GTGGCAGGGCGCTACGAACAATTCTTCCTCTACCTGCTGCAC
rs74315319-R	CATGTGAGATTGGATCTTGCTGGGCTGGCAGATGAGGTAGCAGAG
rs111033313-F	GTGGCAGGGCGCTACGAACAATAGACACAAAATCCCAGTCCCT
rs111033313-R	CATGTGAGATTGGATCTTGCTGGGCAGAGGAACACCACACTCACC
rs121908362-F	GTGGCAGGGCGCTACGAACAATGAGCAATGCGGGTTCTTTGA
rs121908362-R	CATGTGAGATTGGATCTTGCTGGGCCTAGACTTGTGTAATGTTTGCCA
rs26760661-F	GTGGCAGGGCGCTACGAACAATGAGTAGAGTGCTTAGTTGAACAGGG
rs26760661-R	CATGTGAGATTGGATCTTGCTGGGCAAGTGTAAGTTGGGTGCTTTGTG
rs80338939-F	GTGGCAGGGCGCTACGAACAATTCTCCCTGTTCTGTCCTAGC
rs80338939-R	CATGTGAGATTGGATCTTGCTGGGCTGAGCCAGATCTTTCCAATGC
Tri-sites-F	CATGTGAGATTGGATCTTGCTGGGCAAAGGAGGTGTGGGGAGATG
Tri-sites-R	GTGGCAGGGCGCTACGAACAATATGACCCGGAAGAAGATGCT

**Table 2:** Probe sequences.

Probe name	Probe sequences (5′→3′)	5′-Fluorophore	3′-Quencher
rs74315319-P	CTGCTACATTGCCCGACCTACCGAGAA	FAM	BHQ1
rs111033313-P	CATCTTTTGTTTTATTTCAGACGAT	FAM	BHQ1
rs121908362-P	CTTTTTGACGGTCCATGATGCTATAC	ROX	BHQ2
rs267606617-P	ATATAGAGGAGACAAGTCGTAACATGG	HEX	BHQ1
rs267606619-P	CCCGTCACCCTCCTCAAGTATACGGG	Cy5	BHQ3
rs750188782-P	GCAGCCTGGCTGCAGGGTGT	FAM	BHQ1
rs80338943-P	TCAGCTGCAGGGCCCATAGCCGGATGTG	ROX	BHQ2
rs1110333204-P	AACTTCCTCTTCTTCTCGTCTCCGGTA	HEX	BHQ1
rs80338939-P	CAGACGATCCTGGGGGGTGTGAA	Cy5	BHQ3

**Table 3:** *Tm* value obtained from the melting curve identified genotypes.

Mutation site	*Tm* of wild type (°C)	*Tm* of mutant type (°C)	Δ*Tm* (°C)
rs74315319	72	65	7
rs111033313	60	54	6
rs121908362	67	61	6
rs267606617	67	61	6
rs267606619	67	59	8
rs750188782	71	58	13
rs80338943	77	72	5
rs1110333204	63	69	6
rs80338939	72	67	5

**Table 4:** Result of double-blind testing of 268 extracted genomic dna samples.

Mutation site	wt/wt	wt/mu	mu/mu	Total
rs74315319	268 (100%)	0	0	268
rs111033313	255 (95.15%)	9 (3.36%)	4 (1.49%)	268
rs121908362	267 (99.63%)	1 (3.73%)	0	268
rs267606617	265 (98.88%)	0	3 (1.12%)	268
rs267606619	268 (100%)	0	0	268
rs750188782	268 (100%)	0	0	268
rs80338943	236 (88.06%)	29 (10.82%)	3 (1.12%)	268
rs1110333204	259 (96.64%)	9 (3.36%)	0	268
rs80338939	262 (97.76%)	6 (2.24%)	0	268

**Table 5:** Recommended melting conditions on three qPCR platforms.

Platforms	Recommended instrument setting	Measured ramp rate (°C/s)	Total time (min)
LightCycler 96	5 readings/°C	0.05	30
LightCycler 480	5 acquisitions/°C	0.03	45
Bio-Rad CFX-96	Step (0.3°C)/hold (5 s)	0.01	60

**Table 6:** *Tm* values obtained from the different platforms.

Mutation Site	LightCycler 96		LightCycler 480		Bio-Rad CFX-96	
	wt (°C)	mu (°C)	wt (°C)	mu (°C)	wt (°C)	mu (°C)
rs74315319	72	65	72	65	70.5	64
rs111033313	60	54	58	52.5	58	52
rs121908362	67	61	66	60	65	59
rs267606617	67	61	66	61	65	59
rs267606619	67	59	66	58	64.5	57
rs750188782	71	58	69	55	68	54
rs80338943	77	72	76.5	71.5	75	71
rs1110333204	63	69	61.5	67.5	60.5	66.5
rs80338939	72	67	72	67.5	70.5	66.5
